# Bridging prion biology and Alzheimer’s disease: from pathogenic seeds to precision therapeutics

**DOI:** 10.3389/fnmol.2025.1660151

**Published:** 2025-10-22

**Authors:** Wenjin Wang, Zhanhui Feng, Lingfeng Shu, Yongmei Hu, Yuting Chen, Baihui Zhang, Hua Huang

**Affiliations:** ^1^School of Medicine and Life Sciences, Chengdu University of Traditional Chinese Medicine, Chengdu, Sichuan, China; ^2^Guizhou Provincial People’s Hospital, Guiyang, Guizhou, China; ^3^Department of Neurology, Dazhou Central Hospital, Dazhou, Sichuan, China

**Keywords:** Alzheimer’s disease, amyloid-beta, tau protein, prion, conformational propagation, precision intervention

## Abstract

Alzheimer’s disease (AD) is characterized by the pathological aggregation of amyloid-beta (Aβ) and tau proteins, which display self-templating propagation reminiscent of the prion protein (PrP*^Sc^*). Despite these similarities, distinct structural heterogeneities and host interaction mechanisms offer unique avenues for disease-modifying therapies. This review comprehensively synthesizes recent advancements addressing: (1) the conformational commonalities and strain-specificities shared between Aβ/tau and PrP*^Sc^*; (2) the spatiotemporal dissemination patterns of pathogenic seeds within neural networks; and (3) the development of biomarkers and therapeutic strategies rooted in prion theory. By integrating insights from prion biology with AD pathogenesis, we propose a comprehensive “conformation-propagation-microenvironment” framework for precision intervention, thereby offering a novel paradigm to surmount current therapeutic limitations.

## 1 Introduction: a paradigm shift from prion biology to Alzheimer’s disease

### 1.1 The global burden and therapeutic dilemma of Alzheimer’s disease

The global prevalence of dementia is projected to escalate dramatically, from 57.4 million cases in 2019 to an estimated 152.8 million by 2050 (GBD 2019 Dementia Forecasting Collaborators, 2022), according to a 2022 study published in The Lancet Public Health. AD is the primary contributor to this surge, accounting for 60%–80% of all dementia cases. Surveillance data from the United States indicate that AD affects 10.9% (95% CI: 10.5–11.3) of adults aged 65 and older (Alzheimers Dementia, 2024). Between 2000 and 2021, the age-adjusted mortality rate for AD in individuals aged 65 and above increased by over 41% (CDC, 2021). Economic analyses forecast that global medical expenditures related to AD will reach $9.12 trillion by 2050, a 3.6-fold increase from 2030 (Jia et al., 2018). This escalating economic burden underscores the profound impact of AD on patients, families, and healthcare infrastructure.

### 1.2 Limitations of current therapeutic strategies: from the amyloid hypothesis to clinical failures

**FIGURE 1:**
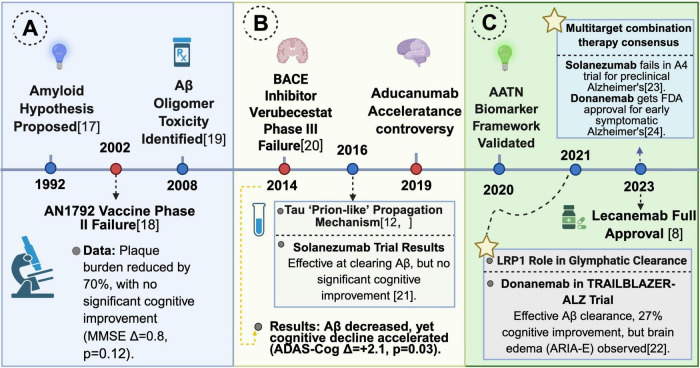
Key milestones and therapeutic advances in Alzheimer’s disease research. **(A)** Early discoveries and clinical trials (1992–2008). This panel highlights the proposal of the Aβ hypothesis (1992) (Hardy and Higgins, 1992), the failure of the AN1792 vaccine (2002) (Orgogozo et al., 2003), and the identification of toxic Aβ oligomers (2008) (Walsh et al., 2002). **(B)** Mechanistic insights and therapeutic challenges (2014–2019). This section covers the failure of the BACE inhibitor Verubecestat in phase III trials (2014) (Egan et al., 2018), the proposal of Aβ’s “prion-like” propagation (2014) (Jucker and Walker, 2018; Shi et al., 2021), and Solanezumab’s lack of cognitive benefit despite Aβ clearance (2016) (Honig et al., 2018). **(C)** Recent breakthroughs and FDA approval (2020–2023). Key events include Donanemab’s efficacy in clearing Aβ plaques with cognitive improvement but associated ARIA-E (2021) (Mintun et al., 2021), characterization of LRP1 receptor involvement in Aβ clearance (2021), Lecanemab’s full FDA approval for early symptomatic AD (2022) (van Dyck et al., 2023), and the outcomes of Solanezumab’s A4 trial and Donanemab’s FDA approval (2023) (Cummings et al., 2023; Sperling et al., 2023). Created in BioRender. Wang, W. (2025) https://BioRender.com/auqjm4v.

For an extended period, the Amyloid Hypothesis, posited by Hardy and Higgins, has been considered the central pathogenic mechanism of AD (Zhang et al., 2023). This hypothesis postulates that Aβ oligomers induce neurotoxicity, subsequently leading to tau hyperphosphorylation, synaptic loss, and cognitive decline (Kurkinen et al., 2023). Therapeutic approaches based on this hypothesis, including anti-Aβ monoclonal antibodies such as Aducanumab and Lecanemab, have demonstrated efficacy in clearing Aβ plaques from the brain. However, their clinical trials have shown limited cognitive benefits. The Phase III trial of Aducanumab demonstrated a modest improvement of merely 0.39 points (out of 18) on the Clinical Dementia Rating-Sum of Boxes (CDR-SB) score in the high-dose group, concurrently associated with a 35% risk of amyloid-related imaging abnormalities-edema (ARIA-E) (Salloway et al., 2022). Despite Lecanemab receiving full FDA approval in 2023, it merely slowed cognitive decline by 27% and was associated with a 12.6% risk of ARIA-E (van Dyck et al., 2023). These findings, as summarized in Figure 1, highlight the inherent limitations of Aβ targeted monotherapy, suggesting a complex, non-linear relationship between Aβ clearance and cognitive improvement.

The intricate pathological landscape of AD further exposes the inadequacies of existing therapeutic strategies. While Aβ deposition is a definitive pathological hallmark of AD, post mortem studies indicate that approximately 30% of cognitively normal elderly individuals exhibit Aβ deposits in their brains (Gjerum et al., 2021). Furthermore, some AD patients, despite negative Aβ PET results, present with characteristic tau propagation patterns (Thijssen et al., 2021), implying that Aβ deposition may serve as an initiating factor rather than the sole driver of disease progression (Chun et al., 2020). The advancement of AD necessitates the synergistic interplay of multiple factors, including Aβ, tau, neuroinflammation, and the microenvironment. Consequently, singular Aβ clearance strategies prove ineffective for patients in advanced stages or those with comorbid pathologies.

### 1.3 A new pathological definition of Alzheimer’s disease: as a protein misfolding disorder

Recent advancements in technologies such as cryo-electron microscopy (cryo-EM) and molecular tracing have significantly deepened our understanding of Aβ and tau protein pathologies in AD. These investigations have revealed that the misfolding and aggregation of Aβ and tau proteins, particularly their soluble oligomeric forms, are primary neurotoxic entities. Critically, they exhibit self-templating propagation characteristics remarkably similar to PrP*^Sc^* (Jucker and Walker, 2018; Shi et al., 2021; Castilla et al., 2008). Although the concept of protein misfolding as a central mechanism in neurodegenerative diseases was extensively explored and proposed by researchers like Soto and Kayed over a decade ago (Kayed et al., 2003; Soto, 2003), these recent breakthroughs have further propelled AD research beyond the traditional amyloid hypothesis toward a more comprehensive paradigm of “protein conformational disease.” The aberrant conformations of Aβ and tau, particularly their cross-β structure, possess the capacity not only for self-replication but also for exerting toxicity through trans-cellular propagation (Peng et al., 2020).

This “prion-like” characteristic implies that once Aβ or tau proteins misfold and adopt a pathogenic conformation, they can function as “seeds,” inducing adjacent normal proteins to misfold and subsequently propagate from one neuron to another, leading to the gradual dissemination of pathology throughout the brain (Manca et al., 2023). It is imperative to note that these pathogenic proteins do not exist in a singular conformation but display considerable conformational heterogeneity, forming diverse misfolded conformations or “strains.” Each distinct strain possesses unique structural attributes, aggregation kinetics, toxicity profiles, and propagation patterns within the brain, which largely dictate the heterogeneous clinical manifestations of the disease (Crowell et al., 2015; Shi et al., 2021; Ferreira and Caughey, 2020; Candelise et al., 2017).

### 1.4 The conformation-propagation-microenvironment (CPM) theory: a unified framework for proteinopathic neurodegeneration

**FIGURE 2:**
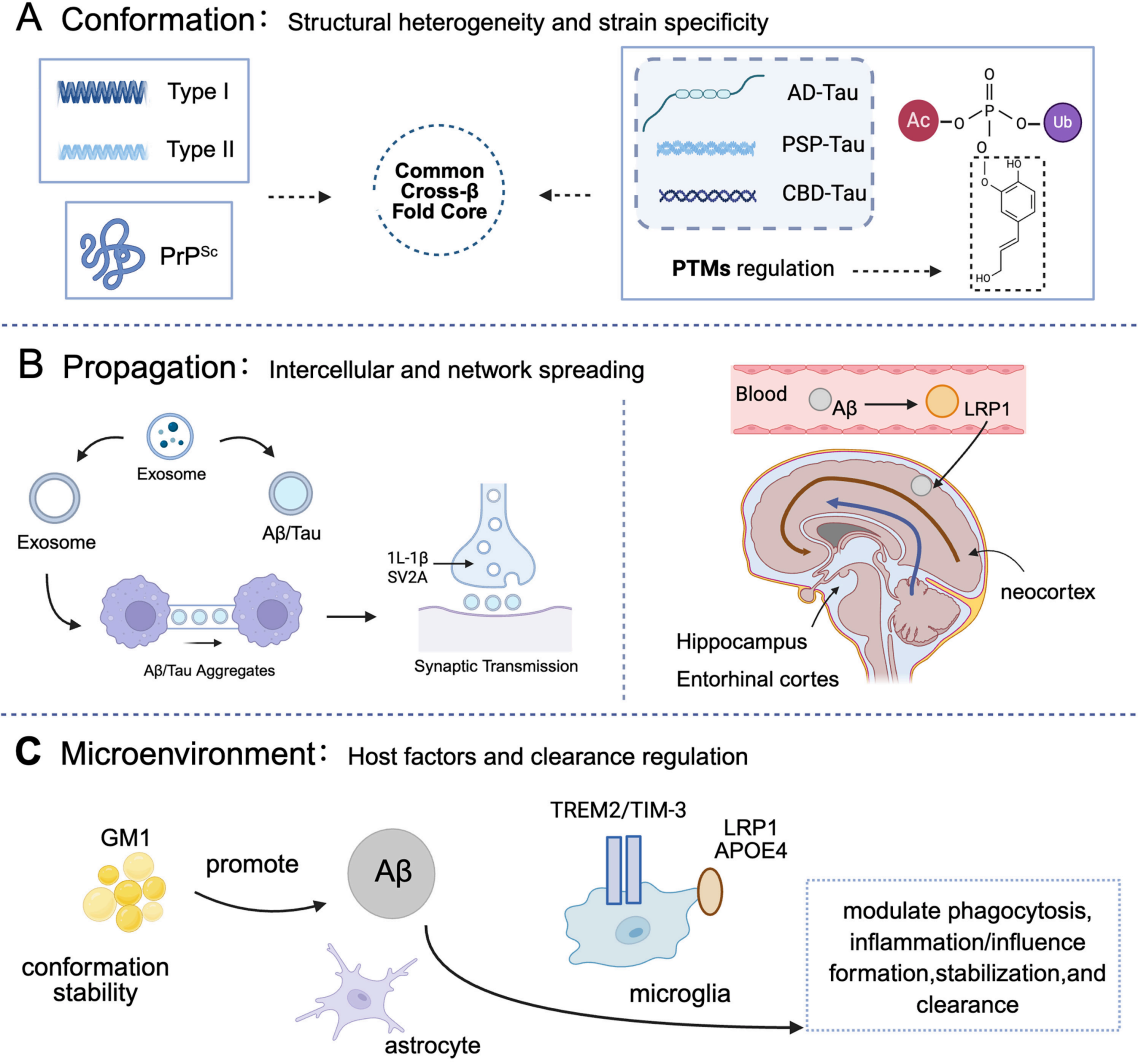
The conformation–propagation–microenvironment (CPM) framework in Alzheimer’s disease. This schematic integrates protein misfolding, spread, and host factors to model disease progression. **(A)** Conformation: pathogenic proteins (Aβ, tau, PrP^Sc^) adopt distinct strains with unique cross-β-sheet structures, influencing propagation and toxicity. Cryo-EM reveals varied fibril architectures (e.g., Aβ42 Types I/II; tau C-shaped folds). PTMs (e.g., phosphorylation) modulate stability and pathogenicity (Ballatore et al., 2007; Bayer, 2022; Buchete and Hummer, 2007; Cabrera et al., 2018; Capocchi et al., 2024; Cummings et al., 2023; Gnoth et al., 2020; Gremer et al., 2017; Grochowska et al., 2017; Hardy and Higgins, 1992; Jucker and Walker, 2018; Kayed et al., 2003; Lauwers et al., 2020; Mamun et al., 2020; Shi et al., 2021; Tarutani et al., 2023a,b; Walsh et al., 2002; Woerman et al., 2016; Zhang et al., 2020). **(B)** Propagation: pathology spreads via exosomes, tunneling nanotubes, and synaptic connections. Tau propagates trans-synaptically along functional networks. Impaired clearance mechanisms (e.g., LRP1 dysfunction) promote Aβ accumulation (Cebecauer et al., 2017; Condello et al., 2020; DeVos et al., 2017; Harrison et al., 2020; Kim et al., 2020; Kong et al., 2021; Li N. et al., 2022; Liu et al., 2017; Malek-Ahmadi et al., 2020; Notario Manzano et al., 2024; P et al., 2022; Pontecorvo and Mintun, 2011; Roemer-Cassiano et al., 2025; Serrano-Pozo et al., 2021; Soliman et al., 2021; Soto et al., 2002; Therriault et al., 2022). **(C)** Microenvironment: host factors such as GM1 ganglioside, LRP1/APOE4, and microglial receptors (e.g.,TREM2) influence aggregation, clearance, and neuroinflammation (Atarashi et al., 2011; Congdon et al., 2023; Desai et al., 2025; Li Y. et al., 2022; Palmqvist et al., 2020; Pontecorvo and Mintun, 2011; Sims et al., 2023; Soto et al., 2002; Tennant et al., 2020). Core concept: misfolded proteins template conformational change in native counterparts, enabling self-replication and disease progression (Jucker and Walker, 2018; Peng et al., 2020; Shi et al., 2021; Soto, 2003). Created in BioRender. Wang, W. (2025) https://BioRender.com/leokh6q.

Acknowledging the limitations of the conventional amyloid hypothesis in elucidating the complex progression mechanisms of AD and the therapeutic impasses of existing strategies (Salloway et al., 2022; van Dyck et al., 2023; Zhang et al., 2023), this review introduces an innovative “Conformation-Propagation Microenvironment” (CPM) theory. This theoretical framework, visually represented in Figure 2, aims to integrate the structural heterogeneity of misfolded proteins, their propagation pathways within the nervous system, and the dynamic influence of the brain microenvironment. Its objective is to provide a cohesive understanding of the initiation, development, and pathological dissemination of protein misfolding diseases, including AD, and to offer a novel paradigm for precise intervention to address the current therapeutic challenges in AD (Dos Santos Nascimento and de Moura, 2024).

The propagation of this neuropathology is not stochastic; rather, it adheres to specific pathways, encompassing intercellular vesicular transport (e.g., exosomes), direct cell-to cell connections (e.g., tunneling nanotubes), and synaptic transmission. Collectively, these propagation routes establish a complex network governing the spatiotemporal dissemination of AD pathology within the brain, a process dynamically modulated by the brain microenvironment.

## 2 Conformation-propagation-microenvironment (CPM) theory: a unified framework for Alzheimer’s disease pathological progression

As delineated in the introduction (Section “1.4 The conformation-propagation-microenvironment (CPM) theory: a unified framework for proteinopathic neurodegeneration”), the intricate pathological progression of AD necessitates a cohesive framework capable of integrating its multifaceted mechanisms. This chapter will meticulously dissect the molecular underpinnings of the three foundational pillars of the CPM theory: the conformation of pathogenic proteins, their propagation within the nervous system, and the dynamic interplay of the brain microenvironment. We will thoroughly examine the implications of these three elements and their reciprocal interactions, elucidating their collective role in driving the pathological cascade of AD, thereby forming a complex, self-perpetuating cycle. Furthermore, we will explore how this theoretical construct, augmented by contemporary research advancements, furnishes a novel theoretical basis for the precise diagnosis and therapeutic intervention of AD.

### 2.1 Conformation: structural heterogeneity and strain specificity of pathogenic seeds

Conformation, the bedrock of the CPM theory, refers to the intrinsic three-dimensional structural characteristics of misfolded proteins, such as Aβ and tau. These proteins manifest considerable conformational heterogeneity in AD, implying the existence of multiple distinct misfolded conformations, or “strains.” Analogous to the diverse strains observed in prions, each of these protein strains may possess unique biological attributes, including distinct aggregation rates, varying degrees of toxicity, differential propagation efficiencies, and specific affinities for particular brain regions (Falcon et al., 2018b; Gremer et al., 2017; Shi et al., 2021). This inherent strain specificity serves as a pivotal molecular determinant for comprehending the pathological diversity and clinical heterogeneity observed in AD. Notably, the conformational features inherent to Aβ and tau exhibit substantial commonalities with the β-helical architecture characteristic of PrP*^Sc^*. Both rely on highly ordered cross-β sheets to maintain their structural integrity and to facilitate self-replication (Shi et al., 2021; Wang et al., 2021). This shared structural principle underpins their “prion-like” behavior and is fundamental to unraveling the complexities of AD pathology.

### 2.2 Conformational polymorphism of Aβ and tau

Cryo-electron microscopy (cryo-EM) investigations have elucidated the existence of multiple conformations of Aβ42 fibrils, exemplified by Type I and Type II variants (Gremer et al., 2017). Similarly, the tau protein displays unique conformations across various tauopathies, including AD, progressive supranuclear palsy (PSP), and corticobasal degeneration (CBD). For instance, AD is characterized by a distinctive C-shaped tau fold, while PSP presents with a twisted helical morphology (Aragão Gomes et al., 2021; Falcon et al., 2018b; Shi et al., 2021; Tarutani et al., 2023a,b; Zhang et al., 2020). These observed conformational distinctions not only influence protein stability but also dictate their interaction profiles with cellular constituents, such as membranes and organelles, thereby modulating their inherent pathogenicity. For example, Aβ Type II fibrils demonstrate a particular propensity to induce oxidative stress and mitochondrial dysfunction (Buchete and Hummer, 2007), a phenomenon potentially attributable to their specific exposed surface areas or charge distribution.

### 2.3 Regulation of conformation by post-translational modifications (PTMs)

Post-translational modifications of proteins, encompassing processes such as phosphorylation, acetylation, truncation, and glycosylation, exert a critical regulatory influence on the conformation of Aβ and tau. For instance, the hyperphosphorylation of tau, particularly at sites like Ser202/Thr205 (AT8 epitope) (Ballatore et al., 2007; Capocchi et al., 2024; Mamun et al., 2020), can precipitate its dissociation from microtubules, thereby fostering misfolding and subsequent aggregation. Conversely, the truncation and pyroglutamylation of Aβ have been shown to augment its seeding activity and aggregation propensity (Bayer, 2022; Cabrera et al., 2018; Gnoth et al., 2020; Grochowska et al., 2017). These PTMs induce alterations in the local structure and charge distribution of proteins, which in turn modulate their folding pathways and the ultimate fibril conformations. Consequently, these modifications profoundly impact the toxicity and propagation capabilities of the proteins. This review posits that specific PTMs may function as “conformational switches,” either inducing or stabilizing highly toxic or highly propagative conformational strains during the nascent stages of the disease.

### 2.4 Conformational commonality with PrP*^Sc^*

The conformational attributes of Aβ and tau exhibit substantial parallels with the β-helical architecture of PrP*^Sc^*. Both protein types rely on highly ordered cross-β sheets to maintain their structural stability and to facilitate self-replication (Shi et al., 2021; Wang et al., 2021). This shared structural principle forms the molecular basis for their observed “prion-like” behavior and is indispensable for a comprehensive understanding of the intricate pathology of AD.

## 3 Structural biology revolution: molecular blueprint of pathogenic seeds and prion-like commonalities

In the preceding section (Section “2 Conformation-propagation-microenvironment (CPM) theory: a unified framework for Alzheimer’s disease pathological progression”), we elucidated the CPM theory, emphasizing the pivotal role of conformational heterogeneity in the pathological progression of AD. Building upon this foundation, the current chapter will meticulously explore the molecular intricacies of “conformation,” a central tenet of the CPM theory. Our focus will be on the structural biology characteristics of Aβ, tau proteins, and PrP*^Sc^*. Recent advancements in cryo-electron microscopy (cryo-EM) technology have profoundly transformed our comprehension of the molecular structures of these pathogenic protein aggregates. These investigations have not only unveiled the atomic-resolution structures of Aβ and tau protein fibrils in AD but, more significantly, have unequivocally demonstrated substantial commonalities in their core structures with PrP*^Sc^*–specifically, the pervasive presence of the cross-β fold structure (Gremer et al., 2017; Jucker and Walker, 2018; Shi et al., 2021). This distinctive structural motif underpins protein misfolding and self-templating propagation, serving as a critical driver of their pathogenicity. This chapter will provide a detailed comparative analysis of the cryo-EM structural features of Aβ, tau, and PrP*^Sc^*, delineating their similarities and differences concerning conformational polymorphism, post-translational modifications (PTMs), and their association with pathogenicity. This endeavor aims to furnish a comprehensive molecular blueprint for deciphering the “prion-like” mechanism of AD.

### 3.1 Aβ and PrP*^Sc^* cross-β fold commonality–conformational polymorphism drives pathogenicity

#### 3.1.1 Polymorphism of Aβ fibrils (type I/type II), differences in membrane toxicity, and structural commonality with PrP*^Sc^*

**FIGURE 3:**
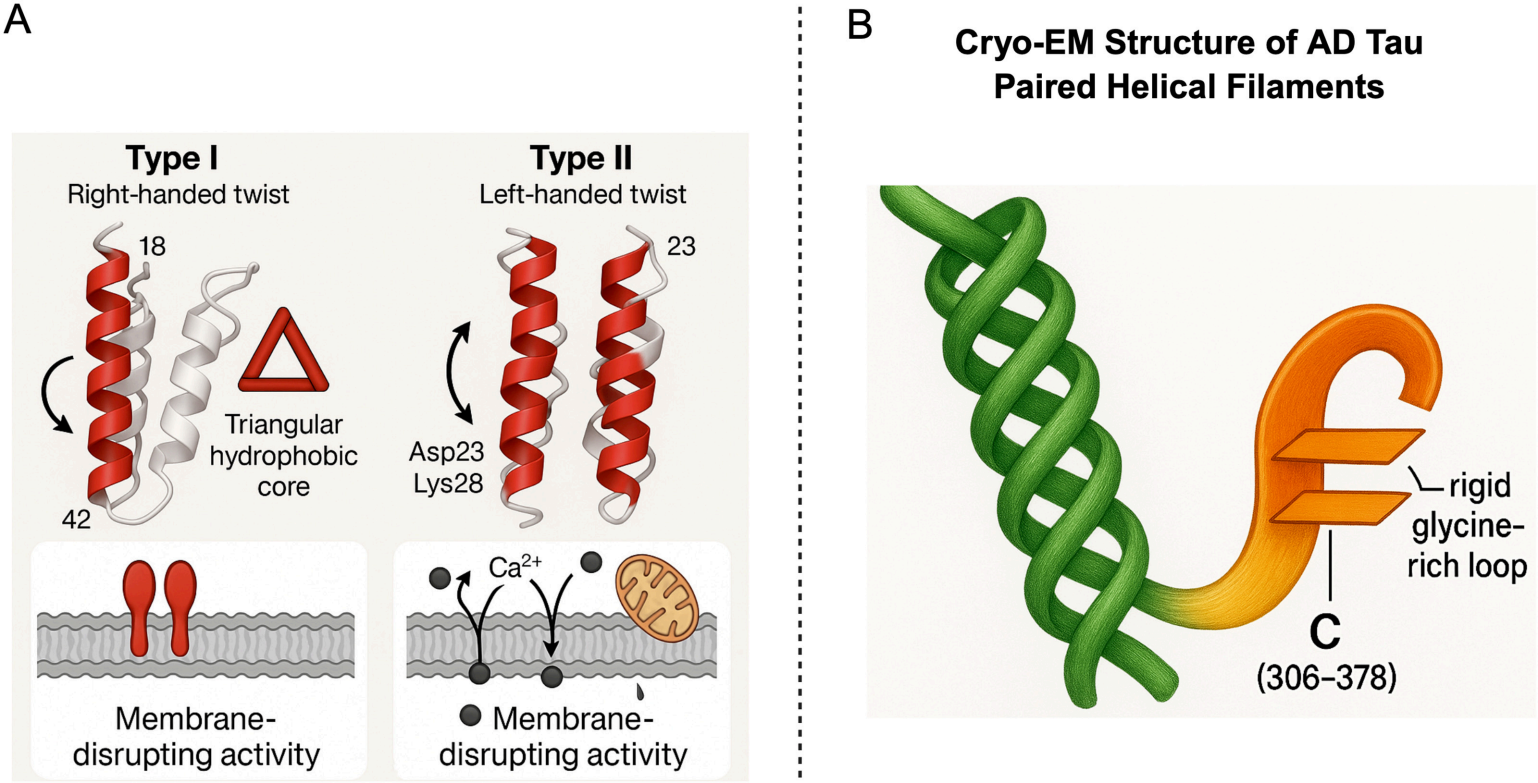
Structural Features of Aβ42 Fibril Polymorphs and Tau Protein. Panel **A** illustrates two main conformations of Aβ42 fibrils-Type I (right-handed twist) and Type II (left-handed twist). These types exhibit distinct core folding patterns (e.g., a triangular hydrophobic core) and specific amino acid residues (such as Asp23 and Lys28), These features lead to different biological activities, primarily membrane disruption. Panel **B** presents the Cryo-EM structure of AD tau, highlighting its paired helical filaments (PHFs) and the unique C-terminal structure (residues 306–378). This structure features parallel β- sheets connected by a glycine-rich loop, offering insights into the molecular mechanisms of tau aggregation in Alzheimer’s disease. Created in BioRender. Wang, W. (2025) https://BioRender.com/m90zv80.

Cryo-electron microscopy studies have unequivocally elucidated two principal structural polymorphs of Aβ42 fibrils: Type I and Type II. These two conformations exhibit marked atomic-level distinctions. Type I fibrils manifest a right-handed helical twist, whereas Type II forms a left handed helical symmetry mediated by salt bridges (Figure 3A; Gremer et al., 2017). Despite their divergent conformations, both share a fundamental cross-β fold structure, a defining characteristic of amyloid proteins. Notably, this cross-β fold structure strikingly resembles the β-helical architecture of the prion protein (PrP*^Sc^*). Both are composed of multiple stacked β-sheets, stabilized by an intricate network of hydrogen bonds, and culminate in highly ordered fibril structures (Shi et al., 2021; Wang et al., 2021). This shared structural foundation elucidates why Aβ can exhibit self-templating propagation properties analogous to PrP*^Sc^*. For instance, Aβ Type II fibrils are particularly prone to inducing oxidative stress and mitochondrial dysfunction (Buchete and Hummer, 2007), a susceptibility potentially linked to their specific exposed surface areas or charge distribution. These characteristics also contribute to the strain specificity observed in PrP*^Sc^* (Crowell et al., 2015).

#### 3.1.2 PrP*^Sc^* β-helical fold and species barrier: manifestation of structural heterogeneity

The pathogenesis of prion diseases fundamentally involves the misfolding of prion protein (PrP) into recalcitrant PrP*^Sc^* isoforms. Cryo-EM investigations have revealed that the pathogenic conformation of PrP*^Sc^* typically adopts a unique β-helical fold structure, characterized by repeating β-sheet units stacked helically (Wang et al., 2021). This β-helical structure parallels the cross-β fold structures of Aβ and tau in its capacity to form stable, pathogenic aggregates. It not only bestows exceptional stability upon PrP*^Sc^* but also dictates its propagation efficiency across diverse species, a phenomenon termed the “species barrier” (Crowell et al., 2015). For example, distinct PrP*^Sc^* strains (e.g., VV1/VV2 types) exhibit variations in β-fold stability, directly influencing their amplification kinetics and pathogenicity. This structural heterogeneity mirrors the polymorphism observed in Aβ and tau, underscoring how subtle conformational disparities can engender significant differences in biological function and disease phenotypic diversity. A more profound understanding of the β-helical structure of PrP*^Sc^* and its variations will enhance our comprehension of the conformational diversity of Aβ and tau in AD and their respective roles in disease progression.

#### 3.1.3 Comparison of transmissibility between prions and Aβ/Tau: similarities, differences, and implications

While Aβ and tau proteins exhibit self-templating propagation properties akin to PrP*^Sc^*, their transmissibility presents notable similarities and distinctions. Classic prions are renowned for their capacity to transmit disease across individuals and even species under experimental conditions, a core pathogenic attribute (Jucker and Walker, 2018). In contrast, the propagation of Aβ and tau primarily manifests as “prion-like” behavior, wherein they disseminate within an individual through self-templating mechanisms between cells and brain regions, typically without involving inter-individual transmission (Buchete and Hummer, 2007).

Experimental evidence indicates that Aβ and tau aggregates can, under specific conditions, induce normal proteins to misfold and form pathological aggregates, leading to pathological dissemination. For example, injecting brain extracts from Alzheimer’s disease patients into mice can induce the propagation of Aβ or tau pathology and neurodegeneration (Tarutani et al., 2023b; Zhang et al., 2020). This “seeding” capability parallels prion propagation (Jucker and Walker, 2018). However, the propagation of Aβ and tau generally lacks the “infectivity” characteristic of classic prions, meaning inter-personal transmission does not occur through routine contact (Lauwers et al., 2020). Studies suggest that the transmission efficiency and host range of Aβ and tau are typically more restricted than PrP*^Sc^*, a limitation potentially attributable to the existence of a “species barrier” and the specificity of distinct protein conformations (Condello et al., 2020; Woerman et al., 2016).

Comprehending these differences in transmissibility holds significant implications for the diagnosis, prevention, and therapeutic strategies of Alzheimer’s disease. Although AD is not contagious, concerns regarding the potential “seeding” risk of Aβ or tau in certain medical procedures necessitate vigilance in the disinfection and handling of medical instruments. In essence, the “prion-like” behavior of Aβ and tau is fundamental to their pathological progression, yet their transmissibility diverges significantly from classic prions. This distinction facilitates a more accurate understanding of AD’s pathological mechanisms and guides future research endeavors.

### 3.2 Tau fibrils: conformational code and association with disease subtypes

#### 3.2.1 Cryo-EM structure and strain specificity of tau fibrils

Abnormal aggregation of tau protein is a defining characteristic of various neurodegenerative diseases, collectively termed tauopathies. Among these, tau pathology in AD possesses distinctive structural features. Cryo-EM structures of AD-specific tau fibrils reveal that their core region forms a unique C-shaped fold (Figure 3B), a structure functionally analogous to the β-helical structure of PrP*^Sc^* in its capacity to drive self templating replication (Aragão Gomes et al., 2021; Fowler et al., 2025). During the formation of this conformation, PTMs of tau protein, particularly acetylation at the K317 site, have been demonstrated to significantly influence the conformation and aggregation properties of tau fibrils (Shi et al., 2021), thereby modulating their toxicity and propagation ability. For a comprehensive exposition on the regulatory mechanisms of PTMs concerning conformational heterogeneity and pathogenicity, please refer to Section “3.4 Post-translational modifications (PTMs): their role in conformational regulation.”

#### 3.2.2 Association of tau subtype structures in PSP/CBD with clinical phenotypes: implications of conformational diversity

Beyond AD, other tauopathies such as PSP and CBD also exhibit unique tau subtype structures, which are intimately linked to their respective clinical manifestations. For instance, tau subtypes in PSP present as twisted helical shapes, whereas those in CBD display a more disordered, prion-like conformation (Aragão Gomes et al., 2021; Tarutani et al., 2023a,b; Zhang et al., 2020). These cryo-EM-elucidated structural diversities further substantiate the principle that “conformation determines function,” implying that distinct misfolded conformations lead to disparate disease phenotypes and propagation characteristics. This conformational diversity strongly aligns with the “strain” concept of PrP*^Sc^*, indicating that the pathological processes of Aβ and tau also manifest similar “strain” phenomena. Different strains may correspond to varying disease progression rates, affected brain regions, and clinical presentations. Identifying these structural features provides crucial insights into disease mechanisms and may guide the development of more precise diagnostic tools and therapeutic modalities targeting specific tauopathy strains.

### 3.3 Comparison of cryo-EM structures of Aβ, Tau, and PrP*^Sc^* fibrils and the molecular basis of the “prion-like” mechanism

**FIGURE 4:**
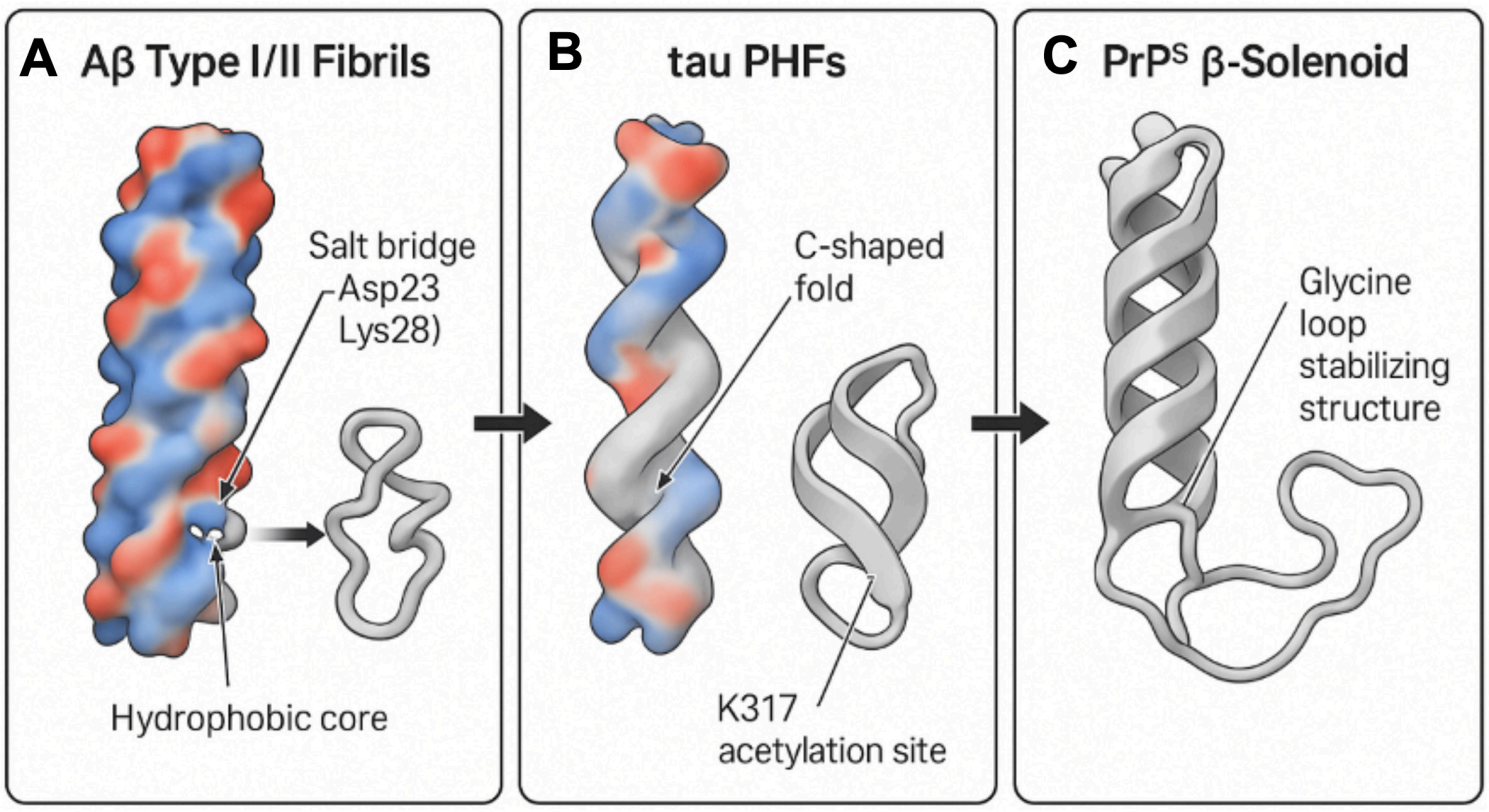
Structural comparison of Aβ, tau, and PrP^Sc^ fibrils using Cryo-EM. This diagram illustrates the structural features of amyloid fibrils from Aβ, tau, and prion proteins (PrP^Sc^) based on Cryo-EM data. **(A)** Aβ type I/II fibrils: these display a hydrophobic core with a crucial salt bridge between Asp23 and Lys28, essential for fibril formation. **(B)** Tau paired helical filaments (PHFs): these exhibit a C-shaped fold and a significant acetylation site at K317, both contributing to fibril structural integrity. **(C)** PrP^Sc^ β-solenoid structure: stabilized by a glycine loop, this structure provides insight into the distinctive folding pattern of prion aggregates. The overall analysis highlights shared yet distinct features that underpin the prion-like mechanisms in neurodegenerative diseases. Created in BioRender. Wang, W. (2025) https://BioRender.com/z4ajr6f.

To visually illustrate the molecular structural commonalities and distinctions among Aβ, tau, and PrP*^Sc^*, and to further elucidate the molecular basis of their “prion-like” propagation, Figure 4 presents representative cryo-EM structures of these three pathogenic protein fibrils. These high-resolution structures are indispensable for comprehending their pathogenicity, conformational polymorphism, and potential therapeutic targets.

Figure 4A depicts the cryo-EM structure of Aβ Type I/II fibrils. The defining characteristic of Aβ fibrils is their highly ordered cross-β fold structure. A hydrophobic core, essential for Aβ fibril stability, is distinctly visible, where β-sheets are tightly packed via hydrophobic interactions. Furthermore, the figure highlights the salt bridge between Asp23 and Lys28, an ionic bond playing a critical role in Aβ fibril formation and stability (Buchete and Hummer, 2007; Gremer et al., 2017). This intricate Aβ structure, particularly its exposed surface features and conformational polymorphism (e.g., Type I and Type II, as detailed in Figure 3A), governs its interaction with cell membranes and its capacity to induce neurotoxicity. This structural specificity suggests that distinct Aβ strains may possess varying pathogenic potentials, analogous to the strain effect observed with PrP*^Sc^*.

Figure 4B presents the cryo-EM structure of tau protein paired helical filaments (PHFs) in AD. The hallmark of AD-tau fibrils is their unique C-shaped fold structure, comprising multiple parallel β-sheets interconnected by glycine-rich loops (Falcon et al., 2018b; Fowler et al., 2025). The figure also explicitly indicates acetylation at the K317 site, a post-translational modification confirmed to significantly enhance the stability of the C-shaped conformation, thereby promoting aberrant tau aggregation and fibrillation. This specific conformation of tau fibrils (as illustrated in Figure 3B) forms the basis for its “prion-like” propagation in AD, enabling it to serve as a template for inducing misfolding of normal tau protein and its dissemination between neurons. Different tauopathies (e.g., PSP, CBD) exhibit distinct conformations of tau fibrils (e.g., twisted helical shape in PSP, disordered conformation in CBD), further emphasizing the critical role of conformational heterogeneity in determining disease phenotypes (Aragão Gomes et al., 2021; Tarutani et al., 2023a,b; Zhang et al., 2020).

Figure 4C illustrates the β-helical structure of prion protein (PrP*^Sc^*). Similar to the cross-β fold of Aβ and tau, the core structure of PrP*^Sc^* also consists of repeating β-sheet units stacked helically, forming a highly stable β-helical structure. The figure specifically denotes the region stabilized by a glycine loop, which is crucial for maintaining the unique folding pattern and stability of PrP*^Sc^* (Wang et al., 2021). This PrP*^Sc^* structure confers extreme resistance to protease degradation and possesses self-replication capabilities, constituting the molecular basis of its infectivity. As underscored in Figure 3, despite overall three dimensional structural differences among Aβ, tau, and PrP^Sc^, they all share a common structural theme–namely, the formation of stable, self-replicating aggregates through highly ordered β-sheet stacking. This shared structural basis is the fundamental reason for their “prion-like” behavior and represents the core molecular connection emphasized in this review, bridging prion biology and Alzheimer’s disease.

### 3.4 Post-translational modifications (PTMs): their role in conformational regulation

Post-translational modifications represent paramount regulatory mechanisms in protein biology. They profoundly alter protein structure, function, stability, localization, and interactions with other molecules through covalent modification of amino acid residues. In AD, aberrant PTMs of Aβ and tau proteins are considered key drivers of their misfolding, aggregation, and propagation, and are intimately linked to disease onset and progression. This section will meticulously examine the primary types of PTMs affecting Aβ and tau proteins, including truncation, pyroglutamylation, and phosphorylation. We will elucidate how these modifications specifically influence the conformational heterogeneity, aggregation kinetics, intercellular propagation ability, and ultimately the neurotoxicity of pathogenic seeds.

#### 3.4.1 Truncation and pyroglutamylation of Aβ promote seed expansion → enhanced seed stability

Post-translational modifications, such as the truncation and pyroglutamylation of Aβ, significantly impact protein aggregation and cytotoxicity. N-terminal truncation of Aβ, in particular, augments its propensity for further aggregation, thereby accelerating the formation of amyloid plaques. Pyroglutamylation, conversely, enhances its seeding activity (Bayer, 2022), indicating a synergistic role for this modification in promoting the amyloid pathology. The influence of these PTMs on Aβ fibril formation and expansion offers valuable therapeutic targets. Inhibiting specific truncation or modification events may mitigate the seeding capacity of Aβ peptides, consequently decelerating the progression of AD.

#### 3.4.2 Tau phosphorylation hotspots drive microtubule dissociation → microtubule dissociation → enhanced propagation ability

Tau phosphorylation constitutes another critical PTM that modulates tau’s functional properties. Specific phosphorylation hotspots, including Ser202/Thr205 (AT8 epitope) (Ballatore et al., 2007; Capocchi et al., 2024), are demonstrably associated with tau dissociation from microtubules. This modification precipitates tau’s detachment from microtubules, subsequently leading to the formation of folded helical filaments and promoting neurodegeneration. Phosphorylation driven microtubule disintegration plays a central role in tau-mediated neurotoxicity. Targeting tau phosphorylation sites through small molecules or gene editing techniques (DeVos et al., 2017; Yang and Qiu, 2024) holds therapeutic promise in tau-related neurodegenerative diseases, such as AD and frontotemporal dementia.

## 4 Spatiotemporal propagation (spreading) mechanisms: from molecules to neural networks

Within the overarching CPM theory, as previously elaborated, “propagation” represents a pivotal process driving the pathological progression of AD. Building upon this framework, the present chapter will meticulously examine the specific “spatiotemporal propagation” of Aβ and tau proteins within the nervous system. A growing body of evidence indicates that the misfolding and intercellular dissemination (spreading) of these pathological proteins exhibit characteristics akin to prions (DeVos et al., 2017; Fowler et al., 2025). This propagation fuels the expansion of pathology across molecular, cellular, and brain network levels. We will delineate the multimodal pathways governing their intercellular transmission and their spatial propagation patterns at the neural network level.

### 4.1 Multimodal pathways of intercellular transmission

**FIGURE 5:**
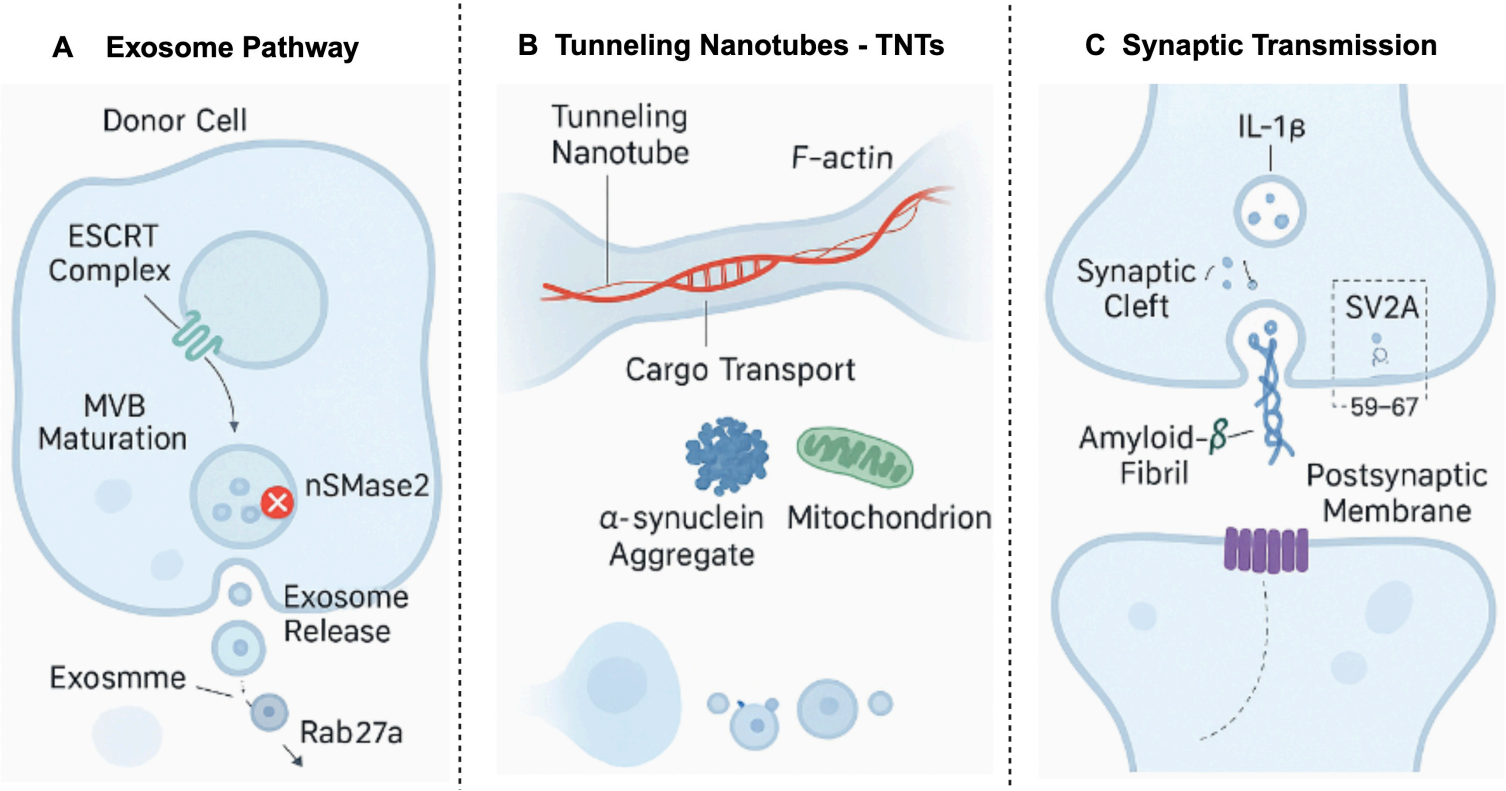
Multimodal pathological seed propagation mechanisms. This figure illustrates three primary mechanisms of pathological seed propagation: **(A)** exosome pathway: the ESCRT complex mediates the maturation of MVBs, with nSMase2 playing a key role in exosome release, and Rab27a regulates exosome transport; **(B)** tunneling nanotubes (TNTs): cargo, including α-synuclein aggregates and mitochondria, is transported through TNTs, driven by F-actin filaments; **(C)** synaptic transmission: amyloid-β fibrils propagate in the synaptic cleft, interacting with SV2A, and influencing the postsynaptic membrane. These pathways highlight the prion-like mechanisms involved in Alzheimer’s disease, providing insight into the spread of pathological seeds within neuronal networks. Created in BioRender. Wang, W. (2025) https://BioRender.com/p7ji4d7.

The intercellular dissemination of pathogenic proteins, including Aβ and tau, relies on a repertoire of molecular mechanisms. As illustrated in Figure 5, exosome-mediated vesicular transport and tunneling nanotube (TNT) transmission are particularly critical among these. These pathways furnish efficient conduits for the horizontal transfer of pathological proteins across cellular boundaries, manifesting features reminiscent of prion-like propagation.

#### 4.1.1 Exosome-mediated Aβ/Tau propagation

Exosomes are nanoscale extracellular vesicles that play a crucial role in intercellular communication (Carretero-González et al., 2018; Mu et al., 2023). In the context of AD, exosomes can encapsulate oligomeric Aβ and hyperphosphorylated tau proteins, facilitating their transfer from compromised neurons to adjacent healthy cells (Soliman et al., 2021). This process promotes the spread of pathological signals, thereby exacerbating synaptic and axonal damage and consequently impairing cognitive function (Li N. et al., 2022). Structural investigations have revealed that Aβ oligomers with exposed hydrophobic regions (e.g., Type II fibril derivatives) exhibit specific binding to GM1 gangliosides on exosomal membranes. This interaction significantly enhances vesicular packaging efficiency, markedly surpassing that observed with hydrophilic conformational strains (Thijssen et al., 2021).

#### 4.1.2 “Prion-like” transmission via tunneling nanotubes (TNTs)

Tunneling nanotubes (TNTs) constitute a class of membranous nanochannels that physically connect adjacent cells, enabling direct cytoplasmic communication, including the transfer of pathogenic protein aggregates (Notario Manzano et al., 2024). Studies have demonstrated that both Aβ and tau can exploit TNTs for intercellular propagation, displaying dissemination characteristics analogous to classic prions. Furthermore, this process is augmented by heightened neuronal activity (Chun et al., 2020; Jucker and Walker, 2018).

#### 4.1.3 Synaptic transmission promotes Aβ and tau propagation

Synapses, serving as vital hubs for information exchange within neural networks, exhibit dysfunctional states that significantly accelerate the propagation of pathological proteins. Upregulation of the inflammatory cytokine interleukin-1 beta (IL-1β) promotes the expansion of TNT networks and exosome release, thereby creating “propagation hotspots.” Concurrently, reduced expression of the synaptic vesicle protein SV2A is correlated with the aberrant accumulation of Aβ and tau, suggesting a substantial role for synaptic transmission and its regulatory molecules in the dissemination of pathological proteins (Kong et al., 2021).

### 4.2 Spatial propagation at the neural network level: Braak staging model

The Braak staging model meticulously delineates the spatiotemporal progression of tau pathology, tracing its dissemination from the entorhinal cortex to the neocortex. This model reveals a non-random, yet anatomically and functionally connected, pathway of propagation among neurons (Therriault et al., 2022).

#### 4.2.1 Trans-synaptic propagation of tau in the default mode network (DMN)

The Default Mode Network (DMN), a critical brain network implicated in memory, selfawareness, and social cognition, demonstrates high susceptibility in AD. Tau pathology preferentially originates in the medial temporal lobe region and subsequently propagates along synaptically connected pathways within the DMN, achieving trans-synaptic “jumping.” This process is guided by the connectome structure of the DMN (Malek-Ahmadi et al., 2020; Roemer-Cassiano et al., 2025). Specifically, C-shaped tau protein originating from the entorhinal layer (Malek-Ahmadi et al., 2020) preferentially binds to heparan sulfate proteoglycans (HSPGs) abundantly present in the DMN (Roemer-Cassiano et al., 2025), facilitating trans-synaptic propagation along hierarchical circuits (entorhinal cortex → hippocampus → neocortex). This hierarchical dissemination defines the Braak staging (Malek-Ahmadi et al., 2020; Therriault et al., 2022). The spatiotemporal characteristics of this process underscore the profound interconnectedness among distinct brain regions.

#### 4.2.2 Aβ clearance impairment and its association with the glymphatic-vascular interface

Aβ protein deposition stems not only from excessive production but also from impaired brain clearance mechanisms, particularly within perivascular regions. Compromised cerebral vascular drainage and blood-brain barrier (BBB) dysfunction exacerbate Aβ deposition (Kim et al., 2020). Specifically, impaired LRP1-mediated Aβ transport across the BBB and diminished glymphatic system function both contribute to increased Aβ accumulation within the brain parenchyma (Harrison et al., 2020; Kim et al., 2020). Apolipoprotein E4 (APOE4) further promotes Aβ enrichment in brain hub regions (e.g., the precuneus) by reducing Aβ hydrodynamic radius (Shi et al., 2021) and compromising meningeal lymphatic vessel structure and function (Peng et al., 2020), thereby accelerating protein homeostasis imbalance. This intricate pathway establishes a positive feedback loop: early DMN hyperactivity augments the synaptic release of tau protein, while compromised Aβ clearance provides a conducive microenvironment for its propagation and accumulation.

### 4.3 Regulation of protein propagation by microenvironmental factors

The microenvironment of the nervous system exerts a profound influence on the initiation and progression of neurodegenerative diseases. Factors such as lipids, the extracellular matrix, and localized inflammatory responses collectively modulate the formation and dissemination of pathological proteins.

#### 4.3.1 GM1 ganglioside promotes conformational stability of Aβ oligomers

GM1 ganglioside anchors the hydrophobic core of Aβ oligomers, thereby promoting their conversion into toxic oligomers (Cebecauer et al., 2017). This interaction extends their extracellular half-life by 2.5-fold and enhances exosomal loading efficiency, consequently increasing Aβ aggregation propensity. This “molecular chaperone” effect offers a promising therapeutic target for intervening in “prion-like” propagation. Specifically, targeting GM1-Aβ binding may provide a viable strategy to modulate the conformational stability of Aβ, thereby disrupting its “prion-like” propagation cascade. Furthermore, lipid raft microdomains also confer protection to phosphorylated tau against phosphatase-mediated degradation (Li N. et al., 2022).

#### 4.3.2 Dual role of LRP1/APOE4 pathway in Aβ clearance and neuroinflammation

Low-density lipoprotein receptor-related protein 1 (LRP1) and Apolipoprotein E4 (APOE4) are pivotal molecules governing Aβ metabolism (Liu et al., 2017; Serrano-Pozo et al., 2021). LRP1 facilitates Aβ transport across the blood-brain barrier and its subsequent degradation (P et al., 2022). Conversely, genetic risk factors such as APOE4 significantly impair LRP1 function, leading to reduced Aβ clearance efficiency and concurrently augmenting central nervous system inflammatory responses (Li Y. et al., 2022). Additionally, the TREM2 R47H mutation compromises microglial phagocytosis of phosphorylated tau, further exacerbating pathological protein accumulation (Buchete and Hummer, 2007). These factors collectively enhance the “prion-like” propagation capacity of pathological proteins, thereby driving the progression of neurodegeneration.

## 5 Pathological seed amplification technologies: a new paradigm for Alzheimer’s disease and prion disease diagnosis

Building upon a profound understanding of the “prion-like” propagation of pathological proteins in AD and prion diseases (Jucker and Walker, 2018; Peng et al., 2020), the concept of misfolded protein aggregates (e.g., PrP^Sc^, Aβ, and tau) as pathogenic “seeds” has opened novel avenues for diagnosing neurodegenerative disorders (Shi et al., 2021). The unique conformational features and strain specificity inherent to these pathological seeds not only drive disease progression but also present opportunities for developing ultrasensitive and highly specific early diagnostic tools (Tennant et al., 2020). While conventional imaging modalities (PET, MRI) and cerebrospinal fluid (CSF) biomarkers [e.g., CSF Aβ42, total tau (t-Tau), phosphorylated tau (p-Tau)] have proven valuable in clinical diagnosis (Gjerum et al., 2021; Pontecorvo and Mintun, 2011; Thijssen et al., 2021), they often exhibit limitations in early disease detection or in differentiating distinct pathological strains. In recent years, the rapid evolution of seed amplification assays (SAAs), exemplified by protein misfolding cyclic amplification (PMCA) and real-time quaking-induced conversion (RT-QuIC), has fundamentally transformed the diagnostic paradigm for prion diseases and AD (Atarashi et al., 2011; Soto et al., 2002). SAAs achieve ultra-early, highly sensitive diagnosis by mimicking the *in vitro* self-replication process of pathological proteins, thereby amplifying minute quantities of pathological seeds to detectable levels (Bizzi et al., 2020; Ito et al., 2012). Furthermore, SAAs can distinguish between various pathological strains by analyzing amplification kinetics and product conformations, offering potential for precise disease subtyping and prognostic assessment (Tarutani et al., 2023a,b). This chapter will meticulously detail the application of PMCA and RT-QuIC SAAs in PrP^Sc^ diagnosis and will particularly focus on the latest advancements in seed amplification reagents for AD diagnosis, including Aβ and tau SAAs. The aim is to elucidate how these technologies translate prion theory into clinically viable diagnostic tools, thereby establishing a new paradigm for early diagnosis, disease monitoring, and the development of precise therapeutic strategies for AD and prion diseases.

### 5.1 Revolutionizing prion disease diagnosis: PMCA and RT-QuIC

Protein misfolding cyclic amplification and RT-QuIC are highly sensitive and specific *in vitro* Seed Amplification Assays (SAAs) (Soto et al., 2002). These techniques replicate the self-templating process of pathogenic proteins *in vivo*, amplifying trace amounts of misfolded proteins (such as PrP^Sc^, β-synuclein, and tau) from biological samples to detectable concentrations (Atarashi et al., 2011; Saborio et al., 2001). Initially developed for the diagnosis of prion diseases (e.g., CJD), these technologies have significantly enhanced the accuracy and timeliness of diagnosis by detecting PrP^Sc^ in bodily fluids like cerebrospinal fluid, with their diagnostic utility being extensively validated (Green, 2019; McGuire et al., 2012). More recently, these techniques have been successfully adapted for diagnostic research in other neurodegenerative disorders, including Parkinson’s disease and Alzheimer’s disease (Orrù et al., 2017).

#### 5.1.1 Protein misfolding cyclic amplification (PMCA)

Protein misfolding cyclic amplification technology, first reported by Saborio et al. (2001), operates on the principle of repeatedly disrupting newly formed PrP^Sc^ aggregates using ultrasound. This process exposes additional “seed” surfaces, thereby accelerating the conversion of normal prion protein (PrPC) to PrP^Sc^. Analogous to the cyclic amplification in polymerase chain reaction (PCR), PMCA can amplify minute quantities of PrP^Sc^ by millions of times within hours to days, reaching detectable levels. The primary advantage of PMCA lies in its exceptional sensitivity, theoretically capable of detecting single PrP^Sc^ molecules. It has been successfully employed for detecting PrP^Sc^ in diverse biological samples, including cerebrospinal fluid, blood, urine, lymphoid tissues, and even skin biopsies (Chen et al., 2024; Pritzkow et al., 2023; Soto et al., 2005). For instance, one study demonstrated PMCA’s efficacy in detecting PrP^Sc^ from skin biopsy samples.

Crucially, Tau-RT-QuIC not only confirms the presence of tau pathology but also differentiates tau strains associated with various tauopathies (e.g., AD, PSP, CBD). Cerebrospinal fluid samples from patients afflicted with distinct tauopathies exhibit unique amplification kinetic curves and product conformations in Tau-RT-QuIC reactions, reflecting the underlying structural disparities of tau protein aggregates across these diseases (Frey et al., 2024; Tarutani et al., 2023a; Wang et al., 2024). For example, the Tau-RT-QuIC amplification curve in AD patients typically displays a distinctive biphasic pattern. The initial lag phase may correlate with the rapid nucleation of AD-specific C shaped tau folds, while the subsequent lag phase might be associated with p-tau396/404- driven fibril elongation. This “conformational fingerprinting” capability enables Tau-RT-QuIC to facilitate precise differential diagnosis between AD and other tauopathies like PSP/CBD, thereby guiding clinicians toward more accurate diagnoses and informed treatment decisions (Lathuiliere and Hyman, 2021).

### 5.2 Application of Aβ seed amplification technology in AD diagnosis

Abnormal aggregation of Aβ protein represents one of the earliest pathological events in AD. Similar to tau, Aβ aggregates possess seeding activity, capable of inducing soluble Aβ monomers to form insoluble amyloid fibrils. Aβ-SAAs, such as Aβ-RT-QuIC or Aβ-PMCA, have been developed for the detection of Aβ seeds in cerebrospinal fluid or blood samples. These technologies enable the detection of extremely low concentrations of Aβ aggregates, thereby providing novel biomarkers for the early diagnosis of AD (Anastasi et al., 2025; Pontecorvo and Mintun, 2011).

Significantly, Aβ-SAAs can also elucidate Aβ conformational features linked to AD risk genes (e.g., APOE4). Studies have revealed that APOE4 carriers typically exhibit faster plasma Aβ amplification rates, a phenomenon potentially attributable to the predominance of compact Aβ conformers induced by APOE4 (Serrano-Pozo et al., 2021). Furthermore, the inclusion of molecules such as GM1 ganglioside in the reaction system can further augment the detection sensitivity of Aβ-SAAs, as GM1 is known to promote the conformational stability of Aβ oligomers (Cebecauer et al., 2017). The integration of Aβ-SAAs with plasma p-Tau217 and Aβ-PET imaging offers the potential to construct a comprehensive “conformation-quantification-localization” diagnostic framework. This integrated approach facilitates a closed loop from molecular conformational characterization to biomarker quantification and subsequent brain pathology localization, thereby translating prion theory into a precise diagnostic tool for AD, and realizing the “conformational feature to clinical decision” pathway.

### 5.3 Challenges and future directions

Despite the considerable promise of seed amplification technologies in the diagnosis of AD and prion diseases, their clinical translation and widespread adoption face several formidable challenges. Foremost among these is the imperative for standardization and rigorous quality control. The reproducibility of results across different laboratories and between various kit batches necessitates further validation. Second, the standardization of sample collection and processing is equally crucial to ensure the integrity and biological activity of pathological seeds within samples. Moreover, in the context of AD, while Aβ and tau SAAs have achieved remarkable progress, the inherent pathological complexity of AD implies that a single biomarker may be insufficient to comprehensively capture the disease’s heterogeneity. Future diagnostic strategies will likely require the integration of multiple biomarkers (including SAAs, imaging, CSF, and blood biomarkers) in conjunction with advanced artificial intelligence and machine learning algorithms to achieve more precise diagnosis and disease subtyping.

## 6 Diagnosis and treatment: from prion theory to clinical translation

With the continuous validation of the “prion-like” paradigm in AD, the traditional treatment model, which primarily focused on end-stage pathology, is progressively being supplanted by strategies that target pathogenic seeds and their inherent conformational heterogeneity. This section integrates the latest advancements in biomarker development, conformation-specific interventions, and brain microenvironment reprogramming, thereby illustrating the clinical translation pathway of prion theory into precision treatment for AD.

### 6.1 Biomarker development: identifying the “molecular fingerprints” of pathogenic seeds

#### 6.1.1 RT-QuIC-based tau strain detection

Real-Time Quaking-Induced Conversion (RT-QuIC) technology demonstrates exceptional advantages in the differential diagnosis of tau pathology. This technique, through the *in vitro* amplification of specific tau protein seeds, enables the differentiation of conformational strains of tau protein across various neurodegenerative diseases (Tennant et al., 2020). Furthermore, RT-QuIC holds considerable promise for detecting early or atypical tau pathology, although its standardization and validation in routine clinical practice remain areas requiring further development.

#### 6.1.2 Comparative analysis of pTau181 and pTau217

Phosphorylated tau protein (p-Tau) serves as a pivotal biomarker for AD, playing a critical role in both disease diagnosis and progression monitoring. For an extended period, pTau181 has been regarded as a classic indicator of AD-specific tau pathology in cerebrospinal fluid, with elevated levels frequently correlating with Aβ deposition and early neurodegeneration (Thijssen et al., 2021). However, in recent years, pTau217 has emerged as a superior next-generation biomarker, exhibiting markedly enhanced diagnostic performance. Research indicates that pTau217 levels can significantly increase even during the preclinical stage of AD, demonstrating a strong correlation with brain Aβ plaque and tau tangle burden. Its sensitivity and specificity surpass those of pTau181 (Gonzalez-Ortiz et al., 2023), particularly in distinguishing AD from other neurodegenerative disorders. Crucially, the detection of pTau217 in plasma also exhibits high accuracy, offering substantial potential for non-invasive early screening of AD (Palmqvist et al., 2020). Consequently, while pTau181 retains its diagnostic utility, pTau217 is progressively becoming the preferred biomarker for early AD diagnosis and monitoring. Their combined application can provide a more comprehensive perspective for precise AD diagnosis.

#### 6.1.3 Complementary application of plasma p-Tau217 and Aβ fibril PET

Phosphorylated tau at site 217 (p-Tau217), as a novel liquid biomarker, demonstrates exceptionally high sensitivity and specificity, outperforming earlier indicators such as p Tau181. Its concentration elevates even prior to observable cognitive decline, reflecting the progression of brain tau pathology. The integration of p-Tau217 with Positron Emission Tomography (PET) imaging technology, which targets Aβ fibrils (Pontecorvo and Mintun, 2011), can establish a multimodal diagnostic framework encompassing quantification and localization. This comprehensive approach provides a robust foundation for the early identification and disease progression tracking in AD. Specifically, the combination of RT-QuIC (for strain identification), p-Tau217 (as a liquid biomarker), and PET (for spatial imaging) constructs a holistic diagnostic strategy based on “conformation-quantification-localization.”

### 6.2 Immunotherapy in Alzheimer’s disease

As our understanding of AD pathogenesis deepens, immunotherapy has emerged as a highly promising therapeutic strategy, primarily aiming to decelerate disease progression through the targeted clearance of pathological proteins. Immunotherapy is broadly categorized into two main approaches: passive immunization and active immunization. These approaches differ significantly in their mechanisms of action, onset of effect, duration, and associated potential risks (Alzheimers Dementia, 2024; GBD 2019 Dementia Forecasting Collaborators, 2022).

#### 6.2.1 Passive immunotherapy

Passive immunotherapy involves the intravenous administration of exogenous monoclonal antibodies (mAbs) designed to specifically bind to and facilitate the clearance of pathogenic proteins. For instance, anti-Aβ monoclonal antibodies such as Aducanumab, Lecanemab, and Donanemab have achieved notable clinical progress. Lecanemab and Donanemab, by selectively targeting Aβ fibrils or plaques, have demonstrably slowed cognitive decline in AD patients, albeit with associated side effects such as amyloid-related imaging abnormalities-edema (ARIA-E) (Sims et al., 2023; van Dyck et al., 2023). While this class of therapies exhibits a rapid onset of action, the exogenous antibodies are subject to metabolic degradation, necessitating periodic re-administration to sustain therapeutic efficacy.

#### 6.2.2 Active immunotherapy

Active immunotherapy primarily stimulates the patient’s endogenous immune system to generate specific antibodies against pathogenic proteins. ACI-35, a tau vaccine developed for AD, aims to elicit a therapeutic effect by inducing the patient’s immune system to produce antibodies specifically targeting phosphorylated tau protein (particularly at the Ser396/404 sites) (Congdon et al., 2023). In contrast to passive immunization, which involves direct antibody injection, ACI-35 stimulates the body to mount an active immune response, thereby potentially offering more durable protection. Although phosphorylation modifications can influence the overall conformation of tau protein, ACI-35 primarily targets this specific post-translational modification rather than the broader three-dimensional aggregated structure of tau protein. Early clinical trials, including the Phase Ib study ISRCTN13033912 (completed in 2017) and the Phase Ib/IIa clinical trial NCT04445831 (conducted in 2022), have confirmed the favorable safety and tolerability profile of ACI-35 in patients with mild to moderate AD, along with its efficacy in inducing robust antibody responses (Congdon et al., 2023).

Immunotherapy represents a novel paradigm for precise intervention in AD. Future research endeavors will focus on optimizing antibody specificity and safety profiles, concurrently exploring combination therapy strategies to achieve more effective pathological clearance and cognitive improvement across various disease stages.

### 6.3 Therapeutic strategies targeting pathogenic seeds: from identification to clearance

#### 6.3.1 Conformation-specific antibodies: Aducanumab, Donanemab

Conformation-specific antibodies constitute a class of antibodies capable of selectively recognizing the unique three-dimensional conformation of a protein, rather than merely its primary amino acid sequence (Desai et al., 2025). In the context of AD treatment, these antibodies primarily target the aberrantly misfolded conformations of Aβ and tau proteins, including their soluble oligomeric/fibrillar forms or tangles (Gaikwad et al., 2024; Zhang et al., 2024). Unlike conventional antibodies that target linear epitopes, conformational antibodies can differentiate between normally folded proteins and pathogenic misfolded proteins, thereby enabling a more precise therapeutic intervention (Desai et al., 2025). Monoclonal antibodies specifically targeting aggregated Aβ or tau conformations are currently undergoing clinical trials and are in the early stages of market introduction. Aducanumab, the first conformation-specific antibody approved for Alzheimer’s disease treatment, recognizes Aβ oligomers and fibrils (Beshir et al., 2022). It has demonstrated some capacity to improve patients’ cognitive function and slow disease progression. However, the discordant results observed in its EMERGE and ENGAGE trials have generated controversy (Budd Haeberlein et al., 2022), underscoring the critical importance of strain adaptability. Furthermore, associated side effects, such as cerebral edema, necessitate further evaluation and optimization. Donanemab, exhibiting superior fibril selectivity, has demonstrated more favorable cognitive maintenance effects and a reduced incidence of ARIA side effects (Reardon, 2023; Sims et al., 2023).

#### 6.3.2 β-sheet breakers: D3 peptide derivatives and polyphenolic compounds

β-sheet breakers achieve non-immune-dependent conformational clearance by disrupting the core structure of pathogenic aggregates (Jani et al., 2021). The D3 peptide and its derivatives can bind complementarily to Aβ fibril structures, inducing their disaggregation. Animal studies have substantiated their efficacy in improving cognitive function (Bocharov et al., 2021; Widera et al., 2014). Polyphenolic compounds, such as Epigallocatechin gallate (EGCG) and curcumin, inhibit Aβ and tau aggregation by interfering with hydrogen bonds and aromatic stacking, concurrently exhibiting notable neuroprotective effects (Li et al., 2024; Xu et al., 2022). Their therapeutic potential in diseases like Alzheimer’s is progressively gaining recognition. While these β-sheet breakers have yielded encouraging results in preclinical investigations, they generally suffer from limitations such as low bioavailability and suboptimal targeting. Nanodelivery systems offer a promising avenue to regulate carriers for targeted drug delivery (Wang et al., 2025), thereby optimizing their pharmacokinetic properties through responsive release platforms.

### 6.4 Microenvironment reprogramming: new pathways to enhance brain clearance efficiency

#### 6.4.1 LRP1 agonists and glymphatic system activation

Microenvironment reprogramming represents a burgeoning therapeutic strategy aimed at augmenting the clearance of pathological protein aggregates by modulating the brain’s immune system and cerebrospinal fluid clearance mechanisms. Low-density lipoprotein receptor-related protein 1 (LRP1), a multifunctional transmembrane receptor, is broadly expressed across various cell types and participates in the endocytosis/signal transduction of multiple ligands and the remodeling of the extracellular matrix (Herz and Bock, 2002). Within the central nervous system, LRP1 regulates Aβ transport across the blood-brain barrier (BBB) and its clearance via the glymphatic system (Ma et al., 2022). Impaired LRP1 function or its downregulated expression in the brains of AD patients is considered a significant contributor to reduced Aβ clearance efficiency, consequently promoting Aβ accumulation and plaque formation in the brain. LRP1 agonists have been shown to enhance CSF Aβ efflux and reduce brain deposition, leading to significant improvements in pathological manifestations in mouse models (Storck et al., 2016). This strategy systemically rectifies the “seeding-clearance” imbalance observed in prion models, offering novel insights for intervention at the seed level.

#### 6.4.2 TREM2 and TIM-3 regulate microglial phagocytosis-inflammation balance

Triggering Receptor Expressed on Myeloid Cells 2 (TREM2) is a microglial surface receptor that modulates their phagocytic capacity for Aβ/tau and their inflammatory responses (Zhong et al., 2019). Activation of TREM2 can promote aggregate clearance while simultaneously suppressing excessive inflammatory responses, thereby mitigating neuroinflammation induced by overactive immune responses (Zhong et al., 2023). Early clinical drug candidates, such as AL002, have demonstrated potential in restoring microglial function and decelerating disease progression (Long et al., 2024). Real-time monitoring of microglial status through functional imaging or fluid biomarkers can guide TREM2-targeted therapy, facilitating individualized immune intervention.

1. T- -cell immunoglobulin and mucin domain-containing protein 3 (TIM-3), an immune checkpoint molecule, plays a significant role in AD. Recent research indicates that in a transgenic mouse model of AD (5 × FAD), the deletion of TIM-3 improved cognitive function and reduced amyloid-beta plaque accumulation. Furthermore, it enhanced phagocytic function and anti-inflammatory gene expression while concurrently reducing pro-inflammatory gene expression (Kimura et al., 2025). TIM-3 is almost exclusively expressed in microglia within the central nervous system, implying that drugs targeting TIM-3 can precisely act on the brain without disrupting the systemic immune system. This research provides a novel therapeutic target for Alzheimer’s disease. For a comprehensive summary of key clinical trial candidates and their mechanisms of action in neurodegenerative diseases, including AD and prion diseases, refer to Table 1.

**TABLE 1:** Key clinical trial candidates for neurodegenerative diseases (AD and prion diseases).

Drug name/code	Disease type	Target/mechanism of action	Clinical trial phase	Key results/status	References
**Alzheimer’s disease (AD) related**					
Lecanemab	AD	Targets Aβ fibrils	Approved	Significantly slows cognitive decline	van Dyck et al., 2023
Donanemab	AD	Targets Aβ plaques	Phase III	Slows cognitive and functional decline	Sims et al., 2023
Aducanumab	AD	Targets Aβ aggregates	Approved	Controversial cognitive improvement, ARIA risk	Budd Haeberlein et al., 2022
ACI-35	AD	Targets phosphorylated tau	Phase Ib/IIa	Good safety, induces antibody response	ClinicalTrials.gov, 2020
Remternetug	AD	Targets Aβ	Phase III	Ongoing	ClinicalTrials.gov, 2025a
Solanezumab	AD	Targets soluble Aβ	Phase III	Did not meet primary endpoint, but some positive signals	Honig et al., 2018; ClinicalTrials.gov, 2019
AAV2-BDNF gene therapy	AD	Brain-derived neurotrophic factor (BDNF)	Phase II/III	Safety being evaluated	ClinicalTrials.gov, 2025b
Gantenerumab	AD	Targets Aβ aggregates	Phase III	Did not meet primary endpoint	ClinicalTrials.gov, 2024
Simufilam	AD	Stabilizes filamin A, reduces pathological Tau	Phase III	Ongoing	Sponsor Cassava Sciences, 2025
**Prion disease related**					
ION717 (PrProfile)	Symptomatic prion disease	Antisense oligonucleotide (ASO), reduces PrP expression	Phase I/IIa	Enrollment complete, evaluating safety and tolerability (first human trial)	Sponsor Ionis Pharmaceuticals, 2024
PRN100	CJD	Anti-PrP monoclonal antibody	Phase I	Observational [patient registry]	University of Zurich, 2023
Quinacrine	sCJD	Small molecule, antimalarial drug	Completed	Did not significantly increase survival rate	ClinicalTrials.gov, 2013
CHARM	Prion disease	Gene editing, inhibits PrP expression	Preclinical (mainly in mouse models)	Over 80% reduction in PrP expression throughout mouse brain	Neumann et al., 2024
Doxycycline	CJD	Antibiotic, may inhibit PrP aggregation	Phase II	Completed, did not significantly extend survival	Haïk et al., 2014

## 7 Challenges and future directions

Despite significant advancements, fundamental questions persist regarding the precise origin and evolution of pathogenic seeds, such as Aβ and tau strains, and their intricate interactions with host neural networks. Future research must delve into how microglia modulate their phagocytic and inflammatory phenotypes across disease stages and the mechanisms by which perivascular spaces influence seed propagation (Jucker and Walker, 2013; Rao et al., 2025; Wodrich et al., 2022). Addressing these requires advanced approaches like spatiotemporally resolved single-cell multiomics technologies. Concurrently, structural biology and computational science are revolutionizing drug discovery. Breakthroughs in cryo-EM provide near-atomic resolution structures of pathogenic protein aggregates, revealing distinct fibril polymorphs (e.g., Aβ42 Types I/II/III, tau PHFs/SFs, PrPSc E196K mutant) that inform targeted drug design (Falcon et al., 2018a; Gilbert et al., 2024; Li et al., 2025; Yang et al., 2022; Zhao et al., 2025). This is powerfully synergized by AI, which accelerates protein structure prediction (e.g., AlphaFold2), virtual screening, and *de novo* drug molecule generation, enabling the identification of compounds that stabilize normal conformations, inhibit misfolding, or promote aggregate clearance (Baek et al., 2021; Jumper et al., 2021; Marra et al., 2025; Popova et al., 2018; Qiu and Cheng, 2024; Thompson et al., 2025; Yang et al., 2019). In clinical translation, the paradigm is shifting toward individualized, multi-dimensional combination therapies within a precision medicine framework. This includes combining agents with different mechanisms, such as Aβ-targeting antibodies (e.g., Lecanemab) with tau vaccines (e.g., ACI-35.030), and stratifying patients based on biomarkers like Aβ-PET, Tau RT-QuIC, and APOE genotype to optimize treatment efficacy (AC Immune, 2021; Novak et al., 2021; Tachibana et al., 2019; van Dyck et al., 2023). However, the clinical translation of seed amplification technologies still faces challenges in standardization and quality control, necessitating robust validation across laboratories. Ultimately, integrating multiple biomarkers with AI and machine learning will be crucial for achieving more precise diagnosis and disease subtyping, paving the way for truly personalized interventions against neurodegenerative disorders.

## 8 Conclusion

This review integrates prion biology with Alzheimer’s disease (AD) pathogenesis, proposing a comprehensive “Conformation-Propagation-Microenvironment” (CPM) framework. We highlight how the structural diversity and strain-specific nature of misfolded proteins, like amyloid-beta (Aβ) and tau, drive AD progression, mirroring prion behavior. Cryo-EM analysis reveals shared cross-β sheet architectures, fundamental to their self-templating and propagation. We detail the spatiotemporal spread of these pathogenic seeds via multimodal pathways (e.g., exosomes, tunneling nanotubes, synaptic transmission), influenced by the brain microenvironment. The review emphasizes the transformative potential of seed amplification technologies (e.g., PMCA, RT-QuIC) for early and precise AD diagnosis, enabling detection and differentiation of protein strains. Furthermore, we discuss emerging therapeutic strategies, including conformation-specific antibodies, β-sheet breakers, and microenvironment modulation. The indispensable roles of Cryo-EM and AI in accelerating targeted drug discovery are also explored. By unifying these insights, the CPM framework offers a new paradigm for understanding AD’s complex etiology and guiding future research toward more effective, personalized diagnostics and therapies, ultimately improving the fight against this devastating neurodegenerative disorder.
